# Risk of Transformation to Acute Myeloid Leukaemia and Myelodysplastic Syndromes in Patients With Myeloproliferative Neoplasms Over Attained Age and Time Since Diagnosis: A Nationwide Cohort Study

**DOI:** 10.1111/ejh.70141

**Published:** 2026-02-20

**Authors:** Nurgul Batyrbekova, Anna Ravn Landtblom, Malin Hultcrantz, Robert Szulkin, Paul W. Dickman, Therese M.‐L. Andersson

**Affiliations:** ^1^ Department of Medical Epidemiology and Biostatistics Karolinska Institutet Stockholm Sweden; ^2^ Clinical Epidemiology Division, Department of Medicine, Solna Karolinska Institutet Stockholm Sweden; ^3^ Department of Medicine, Huddinge Karolinska Institutet Stockholm Sweden; ^4^ Department of Hematology Karolinska University Hospital Stockholm Sweden; ^5^ Department of Medicine, Solna Karolinska Institutet Stockholm Sweden; ^6^ Department of Medicine, Myeloma Service Memorial Sloan Kettering Cancer Center New York New York USA; ^7^ Cytel Stockholm Sweden

**Keywords:** AML, cohort study, disease duration, MDS, myeloproliferative neoplasms

## Abstract

**Background:**

Individuals with polycythemia vera (PV), essential thrombocythemia (ET), and primary myelofibrosis (PMF) face transformation risks to acute myeloid leukemia (AML) and myelodysplastic syndromes (MDS). While older age is linked to increased risk, it remains unclear whether risk increases with age or with disease duration.

**Objectives:**

To examine how age and disease duration affect AML and MDS transformation in MPN.

**Methods:**

We used Swedish nationwide register data to model transformation rates as continuous functions of age and disease duration for each subtype and outcome. Cumulative incidences were estimated accounting for competing risk of death.

**Results:**

This study included 7156 patients with PV, 6810 with ET, and 1080 with PMF diagnosed in 2001–2021. In PV and ET, AML rates increased with disease duration. In PMF, AML rates were highest soon after diagnosis and declined over time. The 10‐year cumulative incidence of AML at diagnosis age 70 was 3.5% in PV, 4.7% in ET, and 18.2% in PMF; for MDS, it was 1.7%, 3.1%, and 10.7%, respectively.

**Conclusion:**

AML and MDS transformation risks vary by MPN subtype and depend on age and disease duration, with disease duration elevating AML risk in PV and ET; incorporating both factors is essential for individualized risk assessment.

## 1 Introduction

Philadelphia chromosome‐negative myeloproliferative neoplasms (MPNs) are chronic bone marrow malignancies characterized by clonal haematopoiesis. The major subtypes are polycythemia vera (PV), essential thrombocythemia (ET), and primary myelofibrosis (PMF) [1]. The clinical course of MPNs is generally indolent, yet patients frequently experience complications including thromboembolic complications, bleeding, infections, and transformation to acute myeloid leukaemia (AML) or myelodysplastic syndromes (MDS) [2, 3, 4, 5, 6, 7].

MPNs can manifest at any age, but are most commonly diagnosed in older individuals, with a median age at diagnosis of around 70 years [8]. The majority of individuals with MPNs can have a long survival spanning decades, but as they age, they may experience more symptoms and an increased risk of transformation to AML or MDS [7, 8]. At 10 years after diagnosis, reported incidences of AML are approximately 2.3% in PV, 0.7%–5% in ET, and 10%–20% in PMF [9, 10, 11, 12, 13, 14]. Prognosis after leukemic transformation is dismal, with median survival of 3–6 months post‐diagnosis, particularly among patients not undergoing allogeneic stem cell transplantation (allo‐SCT) [15, 16].

Several risk factors, depending on subtype, have been linked to post‐MPN AML/MDS such as, older age (≥ 65), leukocytosis (≥ 15 × 10^9^/L), thrombocytosis (platelets > 1000 × 10^9^/L), anemia, thrombocytopenia (< 100 × 10^9^/L), the presence of circulating blasts (≥ 3%) at PMF diagnosis, abnormal karyotype, somatic mutations (e.g., *TP53, IDH1/2, RUNX1, ASXL1, SRSF2, TET2*), and exposure to certain therapies (phosphorus‐32, busulfan, pipobroman) [11, 12, 13, 14, 17, 18, 19, 20, 21, 22, 23, 24]. Genomic mutations and other risk factors inherent to the natural evolution of MPNs tend to accumulate with advancing age and may increase with longer disease duration. However, few studies have systematically examined how the risk of AML/MDS transformation varies simultaneously with age and time since MPN diagnosis in a population‐based setting [2, 25]. To address these parallel timelines, we conducted a nationwide cohort study using Swedish population‐based registers to assess rates of transformation to AML and MDS according to both patient age and time since MPN diagnosis, separately for each subtype.

## 2 Methods

### 2.1 Data Sources and Study Population

Sweden, with a population of approximately 10 million, provides tax‐financed universal healthcare. Each resident is assigned a unique personal identification number, which is consistently recorded across nationwide health and administrative registers, thus enabling individual‐level linkage and long‐term follow‐up.

Since 1958, all malignant cancer diagnoses have been mandatorily reported to the Swedish Cancer Register (SCR), which has high validity and coverage (> 95%) [26]. However, underreporting is recognized for indolent malignancies such as MPNs [27]. Therefore, to improve case ascertainment, we also used the National Patient Register (NPR), which has nationwide coverage of all inpatient hospital discharge diagnoses (since 1987) and all outpatient specialist visits (since 2001), with well‐documented diagnostic validity [28, 29].

We identified all first‐time MPN diagnoses (2001–2021) in the SCR and NPR among individuals aged 18–90 years. For the MPN diagnoses identified through outpatient specialist visits, two separate diagnoses were required, with the second date defined as the diagnosis date. We included PV, ET, and PMF, but excluded patients with MPN‐unclassifiable (MPN‐U) as their first MPN diagnosis. Individuals with a recorded haematological malignancy in the SCR prior to or up to 3 months after the MPN diagnosis were excluded. Patients who emigrated or died within 3 months after diagnosis were also excluded. Information on date and cause of death were obtained from the National Cause of Death Register [30]. The first occurrence of AML and MDS post MPN was identified from the SCR, NPR, and the Cause of Death Register.

Data on cytoreductive drugs (interferon, hydroxyurea, ruxolitinib, anagrelide, and busulfan) among individuals diagnosed between 2006 and 2021 were obtained from the National Prescribed Drug Register [31], a comprehensive nationwide database established in July 2005 that systematically records all prescribed medications that have been dispensed from pharmacies in Sweden.

From the Swedish Blood Cancer Register, established in 2008, we used subtype information for individuals whose MPN was identified in the SCR within 3 months of the diagnosis date in the Blood Cancer Register. Additionally, we obtained patient‐level mutational status of *JAK2*, *CALR*, and *MPL* at diagnosis. Information on specific mutation sites or allele burden was not consistently available. Unfortunately, for patients without a reported mutation in the Blood Cancer Register, it was not possible to determine whether patients lacked the mutation or if it was not assessed.

### 2.2 Statistical Analysis

The index date for the analysis was defined as 3 months after MPN diagnosis. Individuals were followed from the index date until AML or MDS diagnosis, and were censored at emigration, death from causes other than AML/MDS, or end of follow‐up (December 31, 2022), whichever occurred first. For analyses with MDS as the outcome, individuals were additionally censored at AML diagnosis; for analyses with AML as the outcome, MDS events were ignored. We did not censor at allogeneic stem cell transplantation because it was not possible to capture complete transplant data in our register data. This may overestimate transformation risk in PMF, where allo‐SCT is more common, but the overall impact is likely modest given the low frequency of such procedures and their restriction to younger patients [32].

We used flexible parametric survival models on the log‐hazard scale to estimate transformation rates over time since the index date and by attained age simultaneously [33, 34, 35]. Analyses were conducted separately for each MPN subtype and each outcome (AML and MDS). Cumulative incidence functions (CIFs) were estimated for AML and MDS across two timescales, treating death from other causes as a competing event. For MDS transformation, both AML and death from other causes were considered competing events.

To describe exposure to cytoreductive treatment, we conducted two time‐varying analyses. First, we defined a time‐varying indicator to represent whether an individual had a dispensation for a cytoreductive treatment. From the first dispensation of a cytoreductive drug, the individual was assumed to be exposed irrespective of adherence. In the second analysis, we defined a time‐varying variable that captured treatment type and sequence over time. The main interest was to assess how much person‐time was spent in each treatment state: (i) before any cytoreductive treatment; (ii) on or after treatment with interferon without prior exposure to other cytoreductive drugs; (iii) on or after treatment with hydroxyurea without prior exposure to other cytoreductive drugs; (iv) after treatment with both hydroxyurea and interferon but no other cytoreductive drugs; and (v) after treatment with any additional cytoreductive therapy. To reduce misclassification due to short‐term, trial, or non‐initiated prescriptions, we required at least two dispensations before classifying individuals as treated with a specific cytoreductive drug. An exception was made for busulfan, which is typically administered as short courses, and therefore, one dispensation was considered sufficient. These definitions yielded five mutually exclusive person‐time states. Due to the low number of transformation events within treatment categories, both analyses were restricted to descriptive summaries.

Additional analyses were carried out to assess whether transformation rates were different among males and females over time since the index date and by attained age. Further details on the statistical models, Stata code, and all results are available in the interactive Supporting Information online (https://nurbatyr.github.io/Suppl‐material‐transformation‐rates‐in‐MPN‐over‐2ts/). All statistical analyses were conducted using Stata version 18 (StataCorp, 2023) for survival modeling and R version 4.3.1 (R Core Team, 2023) for data management and graphical output.

## 3 Results

We identified 7156 patients with PV, 6810 with ET, and 1080 patients with PMF, diagnosed in 2001–2021 (Table 1). The median age at diagnosis ranged between 68 and 72 years. The median follow‐up time for analyses of AML transformation was 6.1 years for PV, 6.2 years for ET, and 3.7 years for PMF, and for MDS transformation 6.0, 6.1, and 3.5 years, respectively (Table S1). During follow‐up, 190 (2.7%), 191 (2.8%), and 135 (12.5%) transformation events to AML and 115 (1.6%), 166 (2.4%), and 83 (7.7%) transformation events to MDS were observed among patients with PV, ET, and PMF, respectively.

**TABLE 1:** Characteristics of patients diagnosed in Sweden during 2001–2021 with myeloproliferative neoplasms.

	PV (*N* = 7156)	ET (*N* = 6810)	PMF (*N* = 1080)
Sex			
Women	3178 (44.4%)	4200 (61.7%)	452 (41.9%)
Men	3978 (55.6%)	2610 (38.3%)	628 (58.1%)
Age at diagnosis			
Median (Q1, Q3)	70.9 (60.8, 78.2)	68.2 (55.5, 77.3)	71.6 (62.7, 78.7)
18–49	684 (9.6%)	1192 (17.5%)	96 (8.9%)
50–59	1007 (14.1%)	1011 (14.8%)	114 (10.6%)
60–69	1718 (24.0%)	1504 (22.1%)	280 (25.9%)
70–79	2327 (32.5%)	1884 (27.7%)	370 (34.3%)
80–90	1420 (19.8%)	1219 (17.9%)	220 (20.4%)
Calendar period of diagnosis			
2001–2010	3171 (44.3%)	2635 (38.7%)	325 (30.1%)
2011–2021	3985 (55.7%)	4175 (61.3%)	755 (69.9%)

Abbreviations: ET, essential thrombocythemia; PMF, primary myelofibrosis; PV, polycythemia vera.

Among patients diagnosed in 2008–2021, *JAK2* positivity at diagnosis was recorded in 2213 (95%) patients with PV, 1823 (64%) with ET, and 440 (54%) patients with PMF (Table 2). Conversely, *JAK2* status was missing or negative in 112 (5%) PV, 1048 (37%) ET, and 370 (46%) PMF patients. *CALR* positivity was recorded in 249 (9%) with ET and 96 (12%) with PMF, whereas *MPL* positivity was recorded in 71 (3%) with ET and 25 (3%) with PMF; the remaining were listed as either missing or negative.

**TABLE 2:** Characteristics of patients diagnosed with PV, ET or PMF in Sweden in 2008–2021 by somatic mutation status, respectively.

	JAK2 positive	JAK2 negative or not assessed or missing	CALR positive	CALR negative or not assessed or missing	MPL positive	MPL negative or not assessed or missing
PV, *N* = 2325						
*N* patients (% among PV)	2213 (95.2)	112 (4.8)				
Sex						
Female, *n* (%)	1110 (50.2)	58 (51.8)				
Male, *n* (%)	1103 (49.8)	54 (48.2)				
Calendar period of diagnosis						
2008–2015, *n* (%)	1155 (52.2)	62 (55.4)				
2016–2021, *n* (%)	1058 (47.8)	50 (44.6)				
ET, *N* = 2871						
*N* patients (% among ET)	1823 (63.5)	1048 (36.5)	249 (8.7)	2622 (91.3)	71 (2.5)	2800 (97.5)
Sex						
Female, *n* (%)	1110 (60.9)	563 (53.7)	118 (47.4)	1555 (59.3)	41 (57.7)	1632 (58.3)
Male, *n* (%)	713 (39.1)	485 (46.3)	131 (52.6)	1067 (40.7)	30 (42.3)	1168 (41.7)
Calendar period of diagnosis						
2008–2015, *n* (%)	871 (47.8)	587 (56.0)	35 (14.1)	1423 (54.3)	7 (9.9)	1451 (51.8)
2016–2021, *n* (%)	952 (52.2)	461 (44.0)	214 (85.9)	1199 (45.7)	64 (90.1)	1349 (48.2)
PMF, *N* = 810						
*N* patients (% among PMF)	440 (54.3)	370 (45.7)	96 (11.9)	714 (88.1)	25 (3.1)	785 (96.9)
Sex						
Female, *n* (%)	185 (42.0)	162 (43.8)	41 (42.7)	306 (42.9)	15 (60.0)	332 (42.3)
Male, *n* (%)	255 (58.0)	208 (56.2)	55 (57.3)	408 (57.1)	10 (40.0)	453 (57.7)
Calendar period of diagnosis						
2008–2015, *n* (%)	204 (46.4)	196 (53.0)	10 (10.4)	390 (54.6)	< 5	397 (50.6)
2016–2021, *n* (%)	236 (53.6)	174 (47.0)	86 (89.6)	324 (45.4)		388 (49.4)

*Note:* Percentages for sex and calendar period are calculated column‐wise within each mutation category. Patients that do not have positive mutation status could have either missing or negative status as it was not possible to distinguish whether the mutational status was missing, not assessed, or negative.

Overall, 63% of 5566 patients with PV, 71% of 5517 with ET, and 72% of 960 patients with PMF initiated cytoreductive therapy prior to AML/MDS or the end of follow‐up. Approximately 42% of the contributed person‐time at risk (person‐years) in patients with PV, 34% of person‐time in ET, and 33% of person‐time in patients with PMF was spent without cytoreductive therapy. Exposure time to hydroxyurea (≥ 2 dispensations of hydroxyurea) accounted for the largest share of person‐time under cytoreductive therapy: approximately 81% in PV, 77% in ET, and 65% in PMF (Tables S2 and S3). Patients could change treatment group over time, and transformation events within each treatment state were few (72 AML events during hydroxyurea exposure in PV, 68 in ET, and 41 in PMF). Due to the small number of events, analyses by treatment exposure were restricted to descriptive summaries.

We found no strong evidence of differences in AML/MDS transformation rates by sex over time since index date and attained age (data not shown), although limited event counts reduce power to detect differences (Table S4).

### 3.1 Polycythemia Vera

**FIGURE 1:**
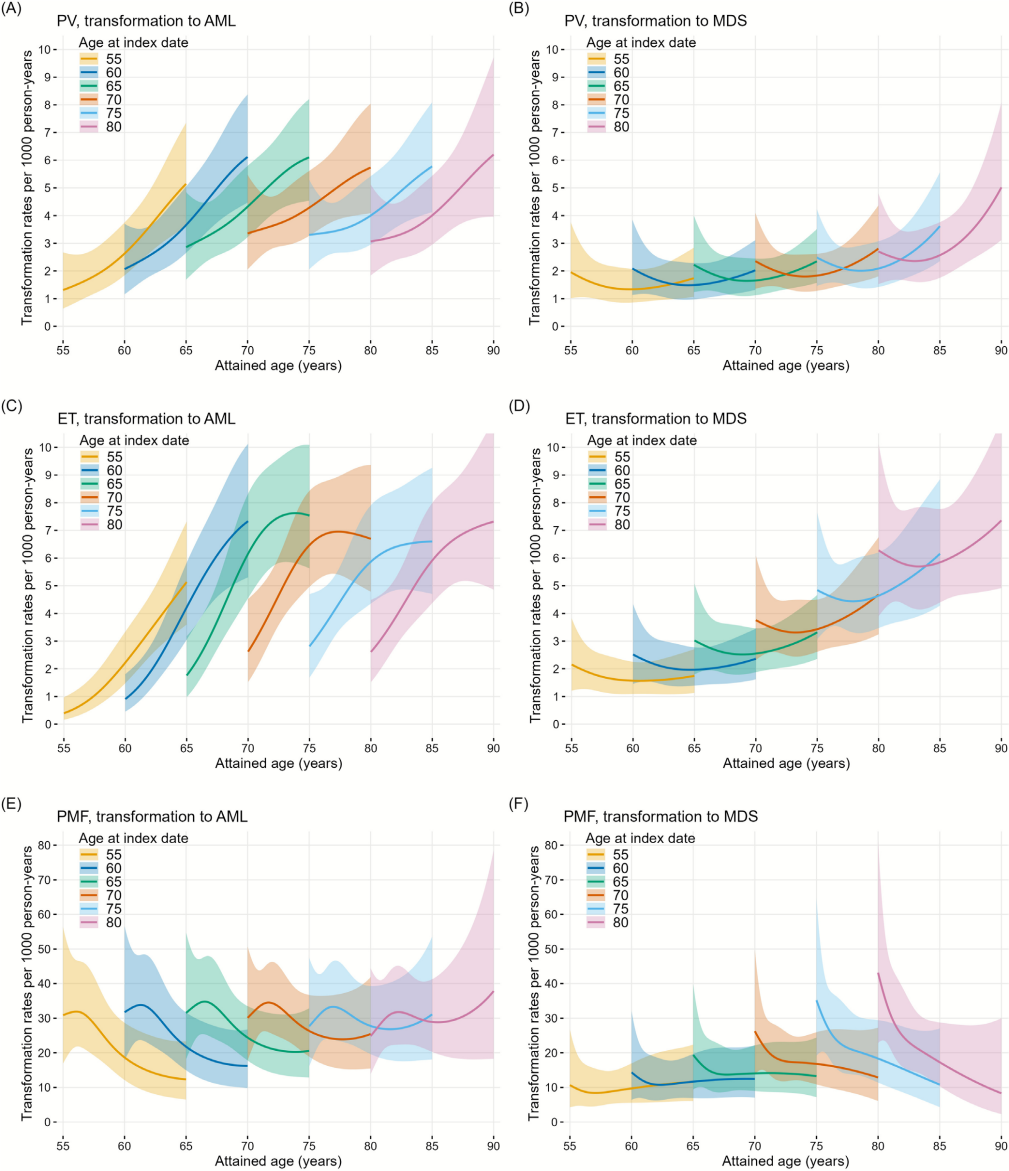
Rates of transformation to AML and MDS per 1000 person‐years over attained age for different ages at the index date for patients with polycythemia vera (PV), essential thrombocythemia (ET), and primary myelofibrosis (PMF), respectively. Index date = MPN diagnosis date + 3 months.

In PV, rates of transformation to AML increased more with time since the index date than with attained age (Figure 1A, Table 3). As a representative example, at attained age 70, the estimated rates per 1000 person‐years with 95% confidence intervals (95% CI) were 3.4 (2.1–5.5) at the index date, 4.3 (3.3–5.6) at 5 years, and 6.1 (4.5–8.4) at 10 years. In contrast, at 5 years since the index date, the corresponding rates (95% CI) at attained ages 65, 75, and 85 were 3.7 (2.7–5.1), 4.3 (3.3–5.6), and 4.0 (2.9–5.4), respectively. MDS transformation rates in PV were lower than those of AML and, unlike AML, increased gradually with attained age (Figure 1B, Table 3).

**TABLE 3:** Estimated rates per 1000 person‐years with 95% confidence intervals (CI) at index date (0 year), 5‐, 10‐, and 15‐years since the index date by subtypes for transformation to AML and to MDS for different ages at the index date, respectively (index date = MPN diagnosis date + 3 m).

Event	Age at index date	Attained age	Time since index date	Estimated rates per 1000 person‐years (95% CI)		
				PV	ET	PMF
AML	55	55	0 year	1.3 (0.6, 2.7)	0.4 (0.2, 1.0)	30.9 (16.9, 56.4)
		60	5 years	2.6 (1.8, 3.8)	2.2 (1.5, 3.3)	18.7 (12.1, 28.7)
		65	10 years	5.1 (3.6, 7.3)	5.1 (3.6, 7.3)	12.3 (6.5, 23.3)
		70	15 years	6.5 (4.4, 9.6)	6.9 (4.6, 10.3)	13.8 (4.7, 40.6)
	60	60	0 year	2.1 (1.2, 3.7)	0.9 (0.5, 1.8)	31.7 (17.8, 56.6)
		65	5 years	3.7 (2.7, 5.0)	4.2 (3.1, 5.8)	21.8 (15.0, 31.7)
		70	10 years	6.1 (4.5, 8.4)	7.3 (5.3, 10.1)	16.2 (9.8, 26.8)
		75	15 years	6.5 (4.5, 9.2)	6.9 (4.8, 9.9)	20.5 (7.5, 56.2)
	65	65	0 year	2.9 (1.7, 4.8)	1.8 (1.0, 3.2)	31.5 (18.2, 54.8)
		70	5 years	4.3 (3.2, 5.8)	6.2 (4.6, 8.3)	24.4 (17.8, 33.5)
		75	10 years	6.1 (4.5, 8.2)	7.5 (5.6, 10.1)	20.6 (12.8, 32.9)
		80	15 years	6.1 (4.1, 9.3)	6.0 (3.9, 9.3)	29.8 (10.6, 83.8)
	70	70	0 year	3.4 (2.1, 5.5)	2.6 (1.5, 4.5)	30.1 (17.9, 50.8)
		75	5 years	4.3 (3.3, 5.6)	6.5 (5.0, 8.4)	26.3 (18.9, 36.7)
		80	10 years	5.7 (4.1, 8.0)	6.7 (4.8, 9.4)	25.4 (15.5, 41.9)
		85	15 years	6.2 (3.9, 9.9)	5.8 (3.5, 9.5)	42.8 (14.0, 130.9)
	75	75	0 year	3.3 (2.1, 5.3)	2.8 (1.7, 4.7)	27.7 (16.1, 47.5)
		80	5 years	4.0 (3.0, 5.4)	5.9 (4.4, 7.9)	27.8 (19.6, 39.3)
		85	10 years	5.8 (4.1, 8.1)	6.6 (4.7, 9.3)	31.1 (18.1, 53.6)
		90	15 years	6.7 (3.7, 12.2)	6.3 (3.4, 11.6)	61.1 (16.5, 225.7)
	80	80	0 year	3.1 (1.8, 5.1)	2.6 (1.5, 4.5)	24.8 (13.9, 44.5)
		85	5 years	4.0 (3.0, 5.4)	5.9 (4.4, 8.0)	28.9 (20.3, 41.3)
		90	10 years	6.2 (4.0, 9.7)	7.3 (4.9, 11.0)	37.9 (18.2, 78.5)
		95	15 years	7.5 (3.1, 17.8)	7.3 (3.1, 17.3)	87.1 (17.7, 428.9)
MDS	55	55	0 year	2.0 (1.0, 3.8)	2.2 (1.2, 3.8)	10.7 (4.2, 26.8)
		60	5 years	1.3 (0.9, 2.1)	1.6 (1.1, 2.3)	9.7 (5.6, 17.0)
		65	10 years	1.7 (1.1, 2.8)	1.7 (1.1, 2.7)	11.6 (6.0, 22.4)
		70	15 years	2.8 (1.7, 4.7)	2.4 (1.5, 4.0)	11.4 (3.2, 40.5)
	60	60	0 year	2.1 (1.1, 3.9)	2.5 (1.5, 4.3)	14.4 (6.4, 32.1)
		65	5 years	1.5 (1.0, 2.3)	2.0 (1.4, 2.8)	11.7 (6.9, 19.7)
		70	10 years	2.0 (1.3, 3.1)	2.4 (1.6, 3.4)	12.5 (7.0, 22.2)
		75	15 years	3.4 (2.2, 5.3)	3.6 (2.3, 5.5)	10.8 (2.7, 42.4)
	65	65	0 year	2.2 (1.2, 4.0)	3.0 (1.8, 5.1)	19.4 (9.4, 39.9)
		70	5 years	1.7 (1.1, 2.4)	2.6 (1.9, 3.5)	14.1 (8.9, 22.1)
		75	10 years	2.3 (1.6, 3.5)	3.3 (2.4, 4.7)	13.3 (7.2, 24.4)
		80	15 years	4.3 (2.7, 6.8)	5.3 (3.4, 8.2)	9.3 (1.9, 45.2)
	70	70	0 year	2.4 (1.4, 4.1)	3.8 (2.3, 6.1)	26.3 (13.8, 49.8)
		75	5 years	1.8 (1.3, 2.6)	3.4 (2.6, 4.5)	16.8 (10.9, 26.0)
		80	10 years	2.8 (1.8, 4.4)	4.7 (3.2, 6.8)	12.9 (6.1, 27.3)
		85	15 years	5.8 (3.6, 9.4)	7.2 (4.5, 11.6)	7.0 (1.1, 43.8)
	75	75	0 year	2.5 (1.5, 4.2)	4.8 (3.1, 7.6)	35.2 (19.1, 64.8)
		80	5 years	2.1 (1.4, 3.1)	4.6 (3.5, 6.2)	18.3 (11.4, 29.5)
		85	10 years	3.6 (2.4, 5.6)	6.2 (4.3, 8.9)	10.8 (4.3, 27.0)
		90	15 years	8.4 (4.8, 14.6)	9.0 (5.2, 15.5)	4.8 (0.5, 44.5)
	80	80	0 year	2.7 (1.5, 4.8)	6.3 (3.9, 10.1)	43.1 (22.8, 81.5)
		85	5 years	2.6 (1.8, 3.8)	5.8 (4.4, 7.8)	17.3 (10.4, 28.8)
		90	10 years	5.0 (3.1, 8.1)	7.4 (4.9, 11.0)	8.3 (2.3, 30.0)
		95	15 years	12.6 (5.6, 28.3)	10.7 (4.8, 23.5)	3.2 (0.2, 50.4)

*Note:* For AML event, death from other causes was considered as a competing event. For MDS event, both transformation to AML and death from other causes were considered as competing events.

Abbreviations: ET, essential thrombocythemia; PMF, primary myelofibrosis; PV = polycythemia vera.

**FIGURE 2:**
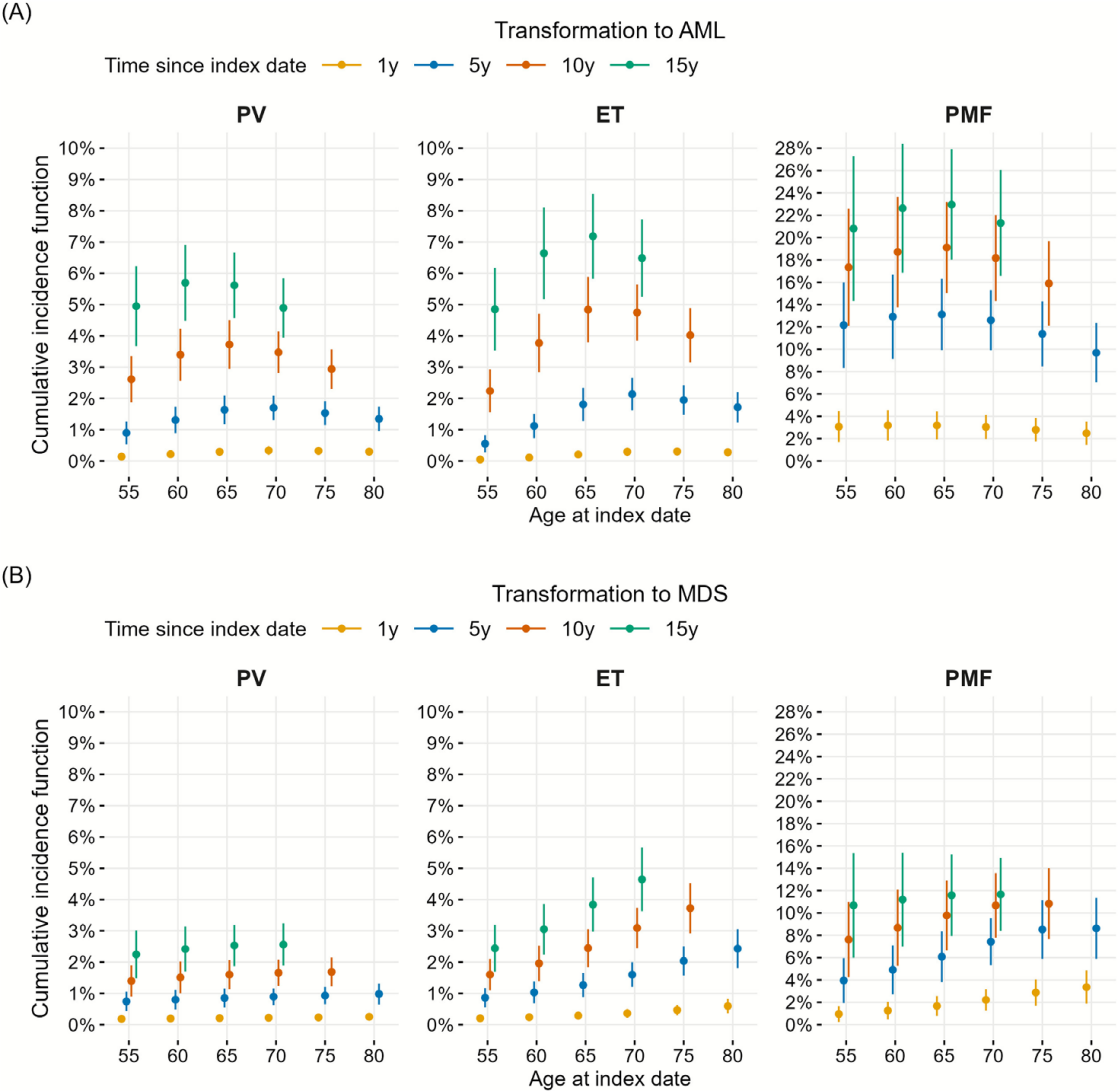
Cumulative incidence at 1‐, 5‐, 10‐, and 15‐years since the index date by MPN subtypes for transformation to AML and to MDS for different ages at the index date, respectively (index date = MPN diagnosis date + 3 months). Panel (A) accounting for death from other causes as a competing event. Panel (B) accounting for transformation to AML or death from other causes as competing events. ET, essential thrombocythemia; PMF, primary myelofibrosis; PV, polycythemia vera.

The estimated cumulative incidence function (CIF) for AML in patients with PV aged 70 at index date, accounting for death from other causes as a competing event, was 0.3% at 1 year, 1.7% at 5 years, 3.5% at 10 years, and 4.9% at 15 years (Figure 2A, Table 4). This corresponds, for example, to 1.7% of patients with PV and index‐age 70 transforming to AML within 5 years. The corresponding 5‐year CIF for MDS in the same patient group, accounting for AML and death as competing events, was 0.9% (Figure 2B, Table 4).

**TABLE 4:** Cumulative incidence functions (CIF) with 95% confidence intervals (CI) at 1‐, 5‐, 10‐, and 15‐years since the index date by subtypes for transformation to AML and to MDS for different ages at the index date, respectively (index date = MPN diagnosis date + 3 m).

Event	Age at index date	Time since index date	PV, CIF % (95% CI)	ET, CIF % (95% CI)	PMF, CIF % (95% CI)
AML	55	1 year	0.1 (0.1, 0.2)	0.0 (0.0, 0.1)	3.1 (1.7, 4.5)
		5 years	0.9 (0.5, 1.3)	0.6 (0.3, 0.8)	12.2 (8.3, 16.0)
		10 years	2.6 (1.9, 3.3)	2.2 (1.6, 2.9)	17.3 (12.1, 22.6)
		15 years	5.0 (3.7, 6.2)	4.8 (3.5, 6.2)	20.8 (14.3, 27.3)
	60	1 year	0.2 (0.1, 0.3)	0.1 (0.0, 0.2)	3.2 (1.8, 4.5)
		5 years	1.3 (0.9, 1.7)	1.1 (0.7, 1.5)	12.9 (9.1, 16.7)
		10 years	3.4 (2.6, 4.2)	3.8 (2.8, 4.7)	18.7 (13.8, 23.7)
		15 years	5.7 (4.5, 6.9)	6.6 (5.2, 8.1)	22.6 (16.9, 28.4)
	65	1 year	0.3 (0.2, 0.4)	0.2 (0.1, 0.3)	3.2 (1.9, 4.4)
		5 years	1.6 (1.2, 2.1)	1.8 (1.3, 2.3)	13.1 (9.9, 16.3)
		10 years	3.7 (2.9, 4.5)	4.8 (3.8, 5.9)	19.1 (15.0, 23.2)
		15 years	5.6 (4.6, 6.7)	7.2 (5.8, 8.5)	23.0 (18.0, 27.9)
	70	1 year	0.3 (0.2, 0.5)	0.3 (0.2, 0.4)	3.0 (2.0, 4.1)
		5 years	1.7 (1.3, 2.1)	2.1 (1.6, 2.7)	12.6 (9.9, 15.3)
		10 years	3.5 (2.8, 4.1)	4.7 (3.9, 5.6)	18.2 (14.3, 22.0)
		15 years	4.9 (3.9, 5.8)	6.5 (5.2, 7.7)	21.3 (16.6, 26.0)
	75	1 year	0.3 (0.2, 0.5)	0.3 (0.2, 0.4)	2.8 (1.7, 3.9)
		5 years	1.5 (1.2, 1.9)	1.9 (1.5, 2.4)	11.4 (8.5, 14.3)
		10 years	2.9 (2.3, 3.6)	4.0 (3.2, 4.9)	15.9 (12.1, 19.7)
	80	1 year	0.3 (0.2, 0.4)	0.3 (0.1, 0.4)	2.5 (1.4, 3.5)
		5 years	1.3 (1.0, 1.7)	1.7 (1.2, 2.2)	9.7 (7.0, 12.3)
MDS	55	1 year	0.2 (0.1, 0.3)	0.2 (0.1, 0.3)	0.9 (0.2, 1.7)
		5 years	0.7 (0.4, 1.0)	0.9 (0.6, 1.2)	4.0 (1.9, 6.0)
		10 years	1.4 (0.9, 1.9)	1.6 (1.1, 2.1)	7.6 (4.3, 11.0)
		15 years	2.2 (1.5, 3.0)	2.4 (1.7, 3.2)	10.7 (6.0, 15.4)
	60	1 year	0.2 (0.1, 0.3)	0.2 (0.1, 0.3)	1.3 (0.5, 2.0)
		5 years	0.8 (0.5, 1.1)	1.0 (0.7, 1.4)	4.9 (2.7, 7.1)
		10 years	1.5 (1.0, 2.0)	2.0 (1.4, 2.5)	8.7 (5.3, 12.1)
		15 years	2.4 (1.7, 3.1)	3.1 (2.2, 3.9)	11.2 (7.0, 15.4)
	65	1 year	0.2 (0.1, 0.3)	0.3 (0.2, 0.4)	1.7 (0.8, 2.6)
		5 years	0.9 (0.5, 1.2)	1.3 (0.9, 1.6)	6.1 (3.8, 8.3)
		10 years	1.6 (1.1, 2.1)	2.4 (1.8, 3.1)	9.8 (6.7, 12.9)
		15 years	2.5 (1.9, 3.2)	3.8 (3.0, 4.7)	11.6 (7.9, 15.2)
	70	1 year	0.2 (0.1, 0.3)	0.4 (0.2, 0.5)	2.2 (1.3, 3.2)
		5 years	0.9 (0.6, 1.2)	1.6 (1.2, 2.0)	7.4 (5.3, 9.5)
		10 years	1.7 (1.2, 2.1)	3.1 (2.4, 3.7)	10.7 (7.8, 13.6)
		15 years	2.6 (1.9, 3.2)	4.6 (3.6, 5.7)	11.7 (8.4, 14.9)
	75	1 year	0.2 (0.1, 0.3)	0.5 (0.3, 0.6)	2.9 (1.7, 4.1)
		5 years	0.9 (0.7, 1.2)	2.0 (1.6, 2.5)	8.5 (5.9, 11.1)
		10 years	1.7 (1.2, 2.1)	3.7 (2.9, 4.5)	10.8 (7.7, 14.0)
	80	1year	0.3 (0.1, 0.4)	0.6 (0.4, 0.8)	3.4 (1.9, 4.8)
		5 years	1.0 (0.6, 1.3)	2.4 (1.8, 3.1)	8.6 (5.9, 11.3)

*Note:* For AML event, death from other causes was considered as a competing event. For MDS event, both transformation to AML, or death from other causes were considered as competing events.

Abbreviations: ET, essential thrombocythemia, PMF, primary myelofibrosis; PV, polycythemia vera.

Both attained age and time since diagnosis had significant associations with AML transformation rates (*p* < 0.05 for each). However, when MDS was the outcome, only attained age showed *p* value < 0.05 in PV (Table S5).

### 3.2 Essential Thrombocythemia

The AML rates in patients with ET increased with both time and attained age (Figure 1C, Table 3). For instance, in patients with attained age 70, the estimated AML rates per 1000 person‐years with 95% CI were 2.6 (1.5–4.5) at the index date, 6.2 (4.6–8.3) at 5 years since the index date, and 7.3 (5.3–10.1) at 10 years since the index date. In individuals with attained ages 65, 75, and 85 at 5 years since the index date, the corresponding rates per 1000 person‐years were 4.2 (3.1–5.8), 6.5 (5.0–8.4), and 5.9 (4.4–8.0), respectively. In contrast, MDS transformation rates in ET increased more with attained age than with time since the index date, though the pattern suggested initially elevated rates followed by a decline and then an increase after approximately 7–10 years (Figure 1D, Table 3).

The estimated CIF for AML among patients with ET with index‐age 70 was 0.3% at 1 year, 2.1% at 5 years, 4.7% at 10 years, and 6.5% at 15 years since the index date (Figure 2A, Table 4). For MDS in the same patient group, accounting for both AML transformation and death as competing events, the corresponding 5‐year CIF was 1.6% (Figure 2B, Table 4).

AML transformation rates in ET varied significantly with both patient age and time since diagnosis (*p* < 0.05 for each). For MDS rates, only attained age showed *p* value < 0.05 (Table S5).

### 3.3 Primary Myelofibrosis

The estimated AML rates in PMF patients were higher than those in PV and ET, and appeared to depend more on time since the index date (Figure 1E, Table 3). Unlike PV and ET, overall, the transformation rates in PMF were elevated at the start of follow‐up, followed by a decline over time. This decline was more pronounced in younger patients than in older patients. Transformation rates to MDS were also higher in PMF than in PV and ET, although consistently lower than the AML rates observed in PMF. MDS rates appeared to depend more on time since the index date, particularly in older patients (Figure 1F, Table 3). The MDS rates were elevated at the start of the follow‐up, especially in older patients (age ≥ 75), and declined over 2 years, reaching a relative plateau by 10 years for all ages.

The cumulative incidence probabilities for AML in PMF were also higher in patients with PMF than in PV and ET (Figure 2A, Table 4). For patients with index‐age 70, the CIF estimates were 3.0% at 1 year, 12.6% at 5 years, 18.2% at 10 years, and 21.3% at 15 years since the index date. The corresponding 5‐year CIF for MDS was 7.4% (Figure 2B, Table 4).

The Wald test of no effects from attained age and time since the index date in PMF yielded *p* value > 0.05 for transformation to AML, whereas for MDS, only attained age showed *p* value < 0.05 (Table S5).

## 4 Discussion

In this largest to‐date population‐based study of patients with MPN, we found that the patterns of transformation to AML and MDS were dependent on both MPN disease duration and attained age. AML rates increased more with time since MPN diagnosis, whereas MDS rates were more dependent on attained age. Moreover, the patterns of how transformation rates changed over time since diagnosis and with age were complex, showing distinct differences between AML and MDS. The dependency of the transformation rates on age, disease duration, or both also differed by subtype, with PMF showing the highest transformation rates to both AML and MDS.

The observation that transformation rates change with time since diagnosis, in addition to attained age, is a novel finding of this study. This temporal dependency suggests that the risk of leukemic transformation is not constant over the disease course but evolves with disease duration and with patient age, differing by subtype and transformation type (AML or MDS). While interpretation should be cautious given the limited number of events, our results support prior studies identifying older age (≥ 65 years) as one of the risk factors [10, 12, 22]. For example, we estimated the 10‐year cumulative incidence of AML in individuals diagnosed at age 70 as 3.5% in PV, 4.7% in ET, and 18.2% in PMF, which are consistent with previously reported 2.3% in PV, 0.7%–5% in ET, and 10%–20% in PMF [9, 10, 11, 12, 13, 14].

In PV and ET, AML transformation rates increased with disease duration, with ET showing higher rates in older patients compared with PV. In PMF, AML rates also showed a stronger dependency on disease duration, but unlike PV and ET, they were elevated at diagnosis and declined over time, particularly in individuals under 65. One possible explanation is that younger patients are more likely to receive allo‐SCT for high‐risk PMF, while those who remain event‐free with longer follow‐up may represent patients with more indolent disease. However, the transformation pattern in PMF was less clear due to the smaller patient population, as reflected by wider confidence intervals.

The rates of transformation to MDS in PV and ET increased more with attained age, especially in ET. The pattern was characterized by elevated rates early after diagnosis, followed by a decline and then a secondary increase after 7–10 years, most pronounced in older patients. In PMF, MDS rates were elevated at the start of follow‐up, particularly in older individuals, but then remained relatively stable across attained age after the first 2 years.

The observed epidemiological patterns in this study align with known underlying biological mechanisms. Leukemic transformation in MPN is largely attributed to the acquisition of mutations and epigenetic alterations, including high‐molecular risk mutations such as *TP53, RUNX1, IDH1/2, SRSF2*, and *ASXL1* [20, 23, 36, 37, 38, 39, 40]. Mutations in *IDH1/2*, more common in PMF, are linked to short‐term transformation, while long‐term transformation has been associated with mutations in *TP53* in ET [23, 40, 41]. A small study (*n* = 49) indicated that leukemic transformation in PV and ET is associated with distinct time‐dependent molecular mechanisms, emphasizing the role of *TP53* mutation in long‐term transformation risk [41]. Together, these findings suggest that mutation‐specific temporal effects on leukemic transformation may underlie the observed pattern of elevated transformation risk early after diagnosis followed by a subsequent decline. Triple‐negative PMF has also been recognized as carrying a particularly high transformation risk [13, 42]. In PMF, as our findings show, leukemic transformation risk is significantly higher closer to the time of diagnosis, whereas in PV and ET it was more dependent on prolonged disease duration and, to some extent, on patient age, which reflects different genomic landscapes and evolution across MPN subtypes. Although we observed sex differences in the transformation rates in PV, ET, and PMF, we found no strong evidence of sex‐specific variation in transformation rates by age or disease duration. However, low event counts limit definitive conclusions.

Transformation to AML/MDS has previously been linked to cytoreductive treatment, particularly with alkylating agents and radioactive phosphorus [43]. In our cohort, hydroxyurea was the most commonly used cytoreductive therapy, reflecting current treatment guidelines that recommend it as first‐line therapy for high‐risk PV and ET. Alkylating agents are now rarely used and typically reserved for later treatment lines due to their suspected leukemogenic potential [44]. Although we provided summary statistics for treatment exposure patterns, the present study was not designed to evaluate treatment‐specific risks, and no conclusions regarding treatment‐related transformation can be drawn.

Our study's population‐based design, covering all diagnosed MPN patients in Sweden from 2001 to 2021, provides an unselected and representative cohort with long‐term follow‐up. Strengths of this study include its large size, nationwide coverage, and high‐quality register‐based follow‐up within a universal healthcare system. This makes our findings generalizable, particularly for a disease with a long and indolent course and supports that previously reported transformation rates [9, 10, 11, 12, 13, 14] are applicable in real‐world settings. However, a limitation is the lack of laboratory data and paucity of reliable genomic information. We could not analyse transformation rates based on recorded mutational status, as it was often unclear whether the absence of such data in some patients indicated that testing had not been performed, results were negative, or the information simply had not been recorded. We also used MPN subtype at diagnosis for analysis, and did not estimate the effect of progression to secondary myelofibrosis in PV and ET on risk of leukemic transformation, since incorporating a time‐varying variable such as progression to secondary myelofibrosis over two timescales was not feasible given the low number of events. Post‐PV myelofibrosis occurs in 12%–21% and post‐ET myelofibrosis in 9%–10% of patients [13], with AML risks comparable to PMF [45]. Slightly higher ET transformation rates compared to PV may also reflect historical misclassification of pre‐PMF as ET before pre‐PMF was recognized as a distinct entity [11, 46, 47]. Diagnostic criteria and clinical practice also evolved over the study period, which may have influenced patient cohort composition over calendar time. Due to sparse data and shorter follow‐up in later years, calendar‐period stratified analyses were not feasible. We were unable to capture accelerated phase disease (10%–19% blasts) due to the lack of a specific diagnostic code, which is clinically important as patients may receive disease‐modifying therapy, including allo‐SCT that directly competes with AML risk. The absence of censoring for allo‐SCT may introduce bias in the transformation estimates, particularly for PMF where transplantation is more common. Reports from the Swedish National Quality Registry for MPN indicate that allo‐SCT represents a small minority of treatment pathways in PMF and is largely confined to younger patients [32]. One possible explanation for the observed transformation patterns in PMF is therefore differential treatment pathways, including referral of younger, high‐risk patients for allo‐SCT. However, we lacked complete individual‐level transplant data to evaluate this directly. Accordingly, estimates for PMF should be interpreted in light of these limitations.

## 5 Conclusion

Transformation to AML and MDS remains a critical complication in the natural history of Philadelphia chromosome‐negative MPNs with varying levels of risk‐prognosis with respect to each of the MPN subtypes. In this largest population‐based study to date, we found that the transformation rates were influenced by both attained age and MPN disease duration. The incidence rates of AML showed a higher association with time since MPN diagnosis while the MDS rates were more dependent on attained age. Moreover, the extent of this dependency and the patterns of transformation differed by subtype, with each subtype showing distinct differences between AML and MDS. This complexity and variation in transformation rates highlight the importance of including age and disease duration in individualized risk assessment and underscore the unmet need for both optimized timing of allogeneic stem cell transplantation as well as new treatment options with true disease modifying features that prevent transformation.

## Author Contributions

Conceptualization: T.M.‐L.A., A.R.L., M.H., and N.B. Data curation: N.B. and T.M.‐L.A. Formal analysis: N.B. and T.M.‐L.A. Funding acquisition: T.M.‐L.A. and M.H. Investigation: N.B. Methodology: N.B. and T.M.‐L.A. Project administration: N.B. Resources: N.B. and M.H. Software: N.B. Supervision: T.M.‐L.A. Validation: N.B. and T.M.‐L.A. Data interpretation: N.B., T.M.‐L.A., A.R.L., M.H., R.S., and P.W.D.. Visualization: N.B. Writing – original draft: N.B. Writing – review and editing: N.B., T.M.‐L.A., A.R.L., M.H., R.S., and P.W.D.. All authors were not precluded from accessing data in the study, and they accept responsibility to submit for publication.

## Funding

The funding sources were the Swedish Cancer Society (grant number: 19 0102, 22 2126), Blodcancerforbundet (grant numbers: 2019‐01965, 2019‐00227, 2023‐02517) and the Memorial Sloan Kettering Core Grant (P30 CA008748). N.B. and R.S. time was funded by Cytel. A.R.L. was funded by the Swedish Blood Cancer Association and by Radiumhemmet. None of the funding sources had any influence on the study design, conduct, and reporting.

## Disclosure

N.B. and R.S. were employees at Cytel when this study was carried out, but Cytel was not involved in the study. M.H. reports research funding from Abbvie, Beigene, BristolMyersSquibb, Daiichi Sankyo, GlaxoSmithKline, Johnson&Johnson, Springworks Therapeutics; and has received honoraria for consultancy/participated in the advisory boards for Curio Science LLC, Intellisphere LLC, Bristol Myers Squibb, Janssen, and GlaxoSmithKline, none related to this study. A.R.L. and T.M.‐L.A. report research funding from MSD, not related to this study. P.W.D. declares no conflict of interest.

## Ethics Statement

This study was conducted in accordance with relevant guidelines and regulations under approval of the Swedish Ethical Review Authority (2020‐05339). The informed consent was not required as we analysed pseudonymised patient data and our analyses did not contain any personal identifiers.

## Conflicts of Interest

The authors declare no conflicts of interest.

## Supporting information


**Table S1:** For each subtype, number of individuals, person‐years, number of events by outcome, respectively.
**Table S2:** Patients with MPN diagnosis in 2006–2021 at different treatment states after index date (3 months post MPN‐diagnosis) during the follow‐up where the outcome is AML. Treatment state is a time‐varying covariate, so patients can go from 0 to 1, from 0 to 2, from 1 to 3, from 2 to 3, and reach state 4 from any state, where ‘0’ = < 2 dispensations of interferon, hydroxyurea, ruxolitinib or anagrelide, and 0 collection of busulfan, ‘1’ = ≥ 2 dispensations of interferon, ‘2’ = ≥ 2 dispensations of hydroxyurea, ‘3’ = ≥ 2 dispensations of each interferon and hydroxyurea, ‘4’ = ≥ 2 dispensations of ruxolitinib or anagrelide, or 1 dispensation of busulfan (with possible previous treatment states 0–3). Starting treatment state can be any of the above.
**Table S3:** Patients with MPN diagnosis in 2006–2021 at different treatment states after index date (3 months post MPN‐diagnosis) during the follow‐up where the outcome is MDS. Treatment state is a time‐varying covariate, so patients can go from 0 to 1, from 0 to 2, from 1 to 3, from 2 to 3, and reach state 4 from any state, where ‘0’ = < 2 dispensations of interferon, hydroxyurea, ruxolitinib or anagrelide, and 0 collection of busulfan, ‘1’ = ≥ 2 dispensations of interferon, ‘2’ = ≥ 2 dispensations of hydroxyurea, ‘3’ = ≥ 2 dispensations of each interferon and hydroxyurea, ‘4’ = ≥ 2 dispensations of ruxolitinib or anagrelide, or 1 dispensation of busulfan (with possible previous treatment states 0–3). Starting treatment state can be any of the above.
**Table S4:** For each subtype, number of individuals, person‐years, number of events by sex and outcome, respectively.
**Table S5:** *p* values from the Wald tests for parameters of t1 and t2 from the model flexible parametric survival model on the log‐hazard scale with two time‐scales: logh = s(t1; γ1) + s(t2; γ2) + s(t1, γ3)⋅(t2, γ4).

## Data Availability

The patient‐level data that support the findings of this study are not publicly available due to regulatory restrictions. However, all the results and Stata code for fitted models and post‐estimation are provided in the Supporting Information online https://nurbatyr.github.io/Suppl‐material‐transformation‐rates‐in‐MPN‐over‐2ts/.
