# Supplementary material for: Risk of Transformation to Acute Myeloid Leukaemia and Myelodysplastic Syndromes in Patients With Myeloproliferative Neoplasms Over Attained Age and Time Since Diagnosis: A Nationwide Cohort Study
Source: Eur J Haematol. 2026 Feb 20;116(6):850–62. doi: 10.1111/ejh.70141 (PMC13141668; doi:10.1111/ejh.70141)
Supplement: Supplementary file 1 — Table S1: For each subtype, number of individuals, person‐years, number of events by outcome, respectively. Table S2: Patients with MPN diagnosis in 2006–2021 at different treatment states after index date (3 months post MPN‐diagnosis) during the follow‐up where the outcome is AML. Treatment state is a time‐varying covariate, so patients can go from 0 to 1, from 0 to 2, from 1 to 3, from 2 to 3, and reach state 4 from any state, where ‘0’ = < 2 dispensations of interferon, hydroxyurea, ruxolitinib or anagrelide, and 0 collection of busulfan, ‘1’ = ≥ 2 dispensations of interferon, ‘2’ = ≥ 2 dispensations of hydroxyurea, ‘3’ = ≥ 2 dispensations of each interferon and hydroxyurea, ‘4’ = ≥ 2 dispensations of ruxolitinib or anagrelide, or 1 dispensation of busulfan (with possible previous treatment states 0–3). Starting treatment state can be any of the above. Table S3: Patients with MPN diagnosis in 2006–2021 at different treatment states after index date (3 months post MPN‐diagnosis) during the follow‐up where the outcome is MDS. Treatment state is a time‐varying covariate, so patients can go from 0 to 1, from 0 to 2, from 1 to 3, from 2 to 3, and reach state 4 from any state, where ‘0’ = < 2 dispensations of interferon, hydroxyurea, ruxolitinib or anagrelide, and 0 collection of busulfan, ‘1’ = ≥ 2 dispensations of interferon, ‘2’ = ≥ 2 dispensations of hydroxyurea, ‘3’ = ≥ 2 dispensations of each interferon and hydroxyurea, ‘4’ = ≥ 2 dispensations of ruxolitinib or anagrelide, or 1 dispensation of busulfan (with possible previous treatment states 0–3). Starting treatment state can be any of the above. Table S4: For each subtype, number of individuals, person‐years, number of events by sex and outcome, respectively. Table S5: p values from the Wald tests for parameters of t1 and t2 from the model flexible parametric survival model on the log‐hazard scale with two time‐scales: logh = s(t1; γ1) + s(t2; γ2) + s(t1, γ3)⋅(t2, γ4). [file EJH-116-850-s001.docx]

# Supplementary material

Full details on the statistical models, Stata code, and all results can be found in the interactive supplementary material online <https://nurbatyr.github.io/Suppl-material-transformation-rates-in-MPN-over-2ts/>.

| **Supplementary Table 1**: For each subtype, number of individuals, person-years, number of events by outcome, respectively. | | | | |
| --- | --- | --- | --- | --- |
| **Event** |  | **PV** | **ET** | **PMF** |
| AML | N | 7,156 | 6,810 | 1,080 |
|  | Person-years | 51,234 | 50,546 | 5,132 |
|  | Events | 190 | 191 | 135 |
|  | Median (Q1, Q3) follow-up time (years) | 6.09 (3.00, 10.22) | 6.18 (3.10, 10.79) | 3.71 (1.75, 6.73) |
| MDS | N | 7,156 | 6,810 | 1,080 |
|  | Person-years | 50,842 | 50,131 | 4,980 |
|  | Events | 115 | 166 | 83 |
|  | Median (Q1, Q3) follow-up time (years) | 6.01 (2.97, 10.13) | 6.12 (3.06, 10.72) | 3.51 (1.57, 6.53) |

**Supplementary Table 2**: Patients with MPN diagnosis in 2006-2021 at different treatment states after index date (3 months post MPN-diagnosis) during the follow-up where the outcome is AML. Treatment state is a time-varying covariate, so patients can go from 0 to 1, from 0 to 2, from 1 to 3, from 2 to 3, and reach state 4 from any state, where '0' = <2 dispensations of interferon, hydroxyurea, ruxolitinib or anagrelide, and 0 collection of busulfan, '1' = ≥2 dispensations of interferon, '2' = ≥2 dispensations of hydroxyurea, '3' = ≥2 dispensations of each interferon and hydroxyurea, '4' = ≥2 dispensations of ruxolitinib or anagrelide, or 1 dispensation of busulfan (with possible previous treatment states 0-3). Note: starting treatment state can be any of the above.

| Subtype | Treatment state | Person-years | Number of events | N |
| --- | --- | --- | --- | --- |
| PV | 0 = <2 dispensations of interferon, hydroxyurea, ruxolitinib or anagrelide, and 0 collection of busulfan | 15,557 | 15 | 4,618 |
|  | 1 = ≥2 dispensations of interferon | 1,366 | <5 | 300 |
|  | 2 = ≥2 dispensations of hydroxyurea | 15,386 | 72 | 2,994 |
|  | 3 = ≥2 dispensations of each interferon and hydroxyurea | 860 | 5 | 203 |
|  | 4 = ≥2 dispensations of ruxolitinib or anagrelide, or 1 dispensation of busulfan (with possible previous treatment states 0-3). | 1,364 | 25 | 386 |
| ET | 0 = <2 dispensations of interferon, hydroxyurea, ruxolitinib or anagrelide, and 0 collection of busulfan | 12,667 | 9 | 4,255 |
|  | 1 = ≥2 dispensations of interferon | 1,519 | <5 | 322 |
|  | 2 = ≥2 dispensations of hydroxyurea | 17,204 | 68 | 3,263 |
|  | 3 = ≥2 dispensations of each interferon and hydroxyurea | 773 | <5 | 180 |
|  | 4 = ≥2 dispensations of ruxolitinib or anagrelide, or 1 dispensation of busulfan (with possible previous treatment states 0-3). | 2,747 | 23 | 585 |
| PMF | 0 = <2 dispensations of interferon, hydroxyurea, ruxolitinib or anagrelide, and 0 collection of busulfan | 1,618 | 45 | 757 |
|  | 1 = ≥2 dispensations of interferon | 253 | <5 | 65 |
|  | 2 = ≥2 dispensations of hydroxyurea | 1,771 | 41 | 481 |
|  | 3 = ≥2 dispensations of each interferon and hydroxyurea | 125 | <5 | 34 |
|  | 4 = ≥2 dispensations of ruxolitinib or anagrelide, or 1 dispensation of busulfan (with possible previous treatment states 0-3). | 586 | 28 | 218 |
| Total number of patients in the cohort: PV N=5566, ET N=5517, PMF N=960. | | | | |

**Supplementary Table 3**: Patients with MPN diagnosis in 2006-2021 at different treatment states after index date (3 months post MPN-diagnosis) during the follow-up where the outcome is MDS. Treatment state is a time-varying covariate, so patients can go from 0 to 1, from 0 to 2, from 1 to 3, from 2 to 3, and reach state 4 from any state, where '0' = <2 dispensations of interferon, hydroxyurea, ruxolitinib or anagrelide, and 0 collection of busulfan, '1' = ≥2 dispensations of interferon, '2' = ≥2 dispensations of hydroxyurea, '3' = ≥2 dispensations of each interferon and hydroxyurea, '4' = ≥2 dispensations of ruxolitinib or anagrelide, or 1 dispensation of busulfan (with possible previous treatment states 0-3). Note: starting treatment state can be any of the above.

| Subtype | Treatment state | Person-years | Number of events | N |
| --- | --- | --- | --- | --- |
| PV | 0 = <2 dispensations of interferon, hydroxyurea, ruxolitinib or anagrelide, and 0 collection of busulfan | 15,511 | 12 | 4,618 |
|  | 1 = ≥2 dispensations of interferon | 1,361 | <5 | 299 |
|  | 2 = ≥2 dispensations of hydroxyurea | 15,289 | 43 | 2,991 |
|  | 3 = ≥2 dispensations of each interferon and hydroxyurea | 858 | <5 | 202 |
|  | 4 = ≥2 dispensations of ruxolitinib or anagrelide, or 1 dispensation of busulfan (with possible previous treatment states 0-3). | 1,330 | 9 | 380 |
| ET | 0 = <2 dispensations of interferon, hydroxyurea, ruxolitinib or anagrelide, and 0 collection of busulfan | 12,617 | 26 | 4,255 |
|  | 1 = ≥2 dispensations of interferon | 1,516 | <5 | 322 |
|  | 2 = ≥2 dispensations of hydroxyurea | 17,100 | 61 | 3,256 |
|  | 3 = ≥2 dispensations of each interferon and hydroxyurea | 772 | <5 | 180 |
|  | 4 = ≥2 dispensations of ruxolitinib or anagrelide, or 1 dispensation of busulfan (with possible previous treatment states 0-3). | 2,711 | 18 | 581 |
| PMF | 0 = <2 dispensations of interferon, hydroxyurea, ruxolitinib or anagrelide, and 0 collection of busulfan | 1,542 | 37 | 757 |
|  | 1 = ≥2 dispensations of interferon | 250 | <5 | 65 |
|  | 2 = ≥2 dispensations of hydroxyurea | 1,736 | 24 | 476 |
|  | 3 = ≥2 dispensations of each interferon and hydroxyurea | 125 | <5 | 34 |
|  | 4 = ≥2 dispensations of ruxolitinib or anagrelide, or 1 dispensation of busulfan (with possible previous treatment states 0-3). | 561 | 11 | 210 |
| Total number of patients in the cohort: PV N=5566, ET N=5517, PMF N=960. | | | | |

| **Supplementary Table 4**: For each subtype, number of individuals, person-years, number of events by sex and outcome, respectively. | | | | | | | |
| --- | --- | --- | --- | --- | --- | --- | --- |
|  | | **PV** | | **ET** | | **PMF** | |
| **Event** |  | **Female** | **Male** | **Female** | **Male** | **Female** | **Male** |
| AML | N | 3,178 | 3,978 | 4,200 | 2,610 | 452 | 628 |
|  | Person-years | 22,557 | 28,677 | 31,713 | 18,832 | 2,265 | 2,867 |
|  | Events | 93 | 97 | 97 | 94 | 56 | 79 |
|  | Median (Q1, Q3) follow-up time (years) | 6.04 (3.06, 9.96) | 6.12 (2.96, 10.41) | 6.35 (3.24, 10.95) | 6.05 (2.95, 10.42) | 4.00 (1.96, 6.88) | 3.48 (1.50, 6.60) |
| MDS | N | 3,178 | 3,978 | 4,200 | 2,610 | 452 | 628 |
|  | Person-years | 22,318 | 28,524 | 31,446 | 18,684 | 2,206 | 2,774 |
|  | Events | 62 | 53 | 94 | 72 | 28 | 55 |
|  | Median (Q1, Q3) follow-up time (years) | 5.94 (3.01, 9.86) | 6.08 (2.93, 10.36) | 6.27 (3.17, 10.90) | 6.01 (2.91, 10.38) | 3.88 (1.91, 6.72) | 3.33 (1.45, 6.24) |

| **Supplementary Table 5**: P-vaues from the Wald tests for parameters of t1 and t2 from the model flexible parametric survival model on the log-hazard scale with two time-scales: logh=s(t1;γ1)+s(t2;γ2)+s(t1,γ3)⋅(t2,γ4). | | | | |
| --- | --- | --- | --- | --- |
|  | **AML** | | **MDS** | |
|  | **t1 (time since index date)** | **t2 (attained age)** | **t1 (time since index date)** | **t2 (attained age)** |
| PV | 0.02702 | 0.00427 | 0.07651 | 0.00179 |
| ET | 0.00790 | 0.00000 | 0.48399 | 0.00000 |
| PMF | 0.07767 | 0.09285 | 0.11817 | 0.03222 |
